# Association of PTH and vitamin D-related genes with dental development in Brazilian children: a cross-sectional study

**DOI:** 10.1590/1807-3107bor-2025.vol39.033

**Published:** 2025-03-31

**Authors:** Isabela Ribeiro Madalena, Erika Calvano Küchler, Caio Luiz Bitencourt Reis, Mirian Aiko Nakane Matsumoto, Maria Bernadete Sasso Stuani, Thaís Vilalba Paniagua Machado do Nascimento, Christian Kirschneck, Flares Baratto-Filho, Maria Angélica Hueb de Menezes-Oliveira, César Penazzo Lepri

**Affiliations:** (a) Universidade de Uberaba – Uniube, School of Dentistry, Department of Biomaterials, Uberaba, MG, Brazil.; (b) University Hospital Bonn, School of Medicine, Department of Orthodontics, Bonn, Germany.; (c) Universidade de São Paulo – USP, School of Dentistry of Ribeirão Preto, Department of Pediatric Dentistry, Ribeirão Preto, SP, Brazil.; (d) Universidade Tuiuti do Paraná – UTP, School of Dentistry, Curitiba, PR, Brazil.

**Keywords:** Odontogenesis, Parathyroid Hormone, Vitamin D, Genes

## Abstract

The aim was to evaluate the association between dental development (dental maturity) and genetic polymorphisms in *PTH* and genes involved in vitamin D synthesis in a cohort of Brazilian children. This retrospective cross-sectional study was performed on children receiving orthodontic treatment. Patients who had already undergone orthodontic treatment previously, those with syndromes, congenital anomalies, craniofacial deformities, and those with a previous history of dental trauma and bilateral agenesis/missing tooth/teeth were excluded. Panoramic radiographs were used for dental age evaluation according to the method proposed by Demirjian, Goldstein, and Tanner (1976). A delta [dental age minus chronological age (DA-CA)] was calculated to determine whether the patient's dental age was normal, delayed (negative values), or advanced (positive values). DNA isolated from buccal cells was used for genotyping genetic polymorphisms in *PTH* (rs694, rs307247, and rs6256), *VDR* (rs7975232), *CYP27B1* (rs464653), and *CYP24A1* (rs927650). A statistical analysis was performed and p<0.05 indicated statistical difference. A total of 79 orthodontic patients were included (44 (55.70%) girls and 35 (44.30%) boys). Demirjian, Goldstein, and Tanner's method (1976) overestimated the age of patients by 0.75 years. None of the genetic polymorphisms were associated with dental age (p>0.05). In conclusion, there is no association between genetic polymorphisms in *PTH* and genes involved in vitamin D synthesis and dental maturity.

## Introduction

Dental development (tooth formation) is extremely important to maintain the balance within the stomatognathic system. Dental development is used for forensic identification and as an indicator of maturation in clinical practice.^1,2^ In 1973, Demirjian, Goldstein, and Tanner introduced a method that estimates biological age based on dental development in the seven left mandibular teeth, categorizing their stages of calcification from A to H.^3^ Although the method of Demirjian, Goldstein, and Tanner (1973) was designed for French-Canadian populations,^4,5^ it is widely applied in different populations,^5,6^ including Brazilian children.^5-7^ This method has been applied to evaluate dental age variability in different populations and estimate possible associations between different traits and delayed or advanced dental development.^1,5,7^ Dental development is a multilevel, multidimensional, long-lasting, progressive, and complex process,^8^ in which local, systemic, environmental, and genetic factors play an important role.^9-11^ The understanding of the molecular mechanisms involved in dental development is crucial in health sciences, however, the molecular and genetic factors involved in dental age variability are still poorly explored.^5,7^

Two essential hormones for bone and dental development are parathyroid hormone (*PTH*)^12^ and vitamin D.^13^ PTH regulates mineral ion homeostasis, skeletal development, and bone renewal by activating parathyroid hormone receptor 1 (*PTH1R*). In the kidneys, PTH stimulates the expression of 25-hydroxyvitamin D_3_ (25(OH)D_3_), which is the best indicator of serum levels of vitamin D in the human body.^14-16^ Vitamin D is a secosteroid hormone, which plays an important role in calcium homeostasis and is vital for tissue mineralization.^17^ Although it is stimulated by *PTH*, vitamin D is obtained from exposure of the skin to UVB radiation from sunlight. The biological effects of vitamin D are mediated by its binding to the intracellular receptor, the vitamin D receptor (*VDR*), in addition to other important genes such *CYP27B1*(Cytochrome P450 Family 27 Subfamily B Member 1) and *CYP24A* (Cytochrome P450 Family 24 Subfamily A Member 1).^18^ Some studies have suggested an association of vitamin D with craniofacial development^19-21^ and dental development.^19,22-24^

Our hypothesis in the present study is that genetic polymorphisms in the gene encoding *PTH* and the vitamin D-related genes may be involved in the delay or acceleration of dental development. Genetic polymorphisms in *PTH*, *VDR*, *CYP24A,* and *CYP27B1* were selected based on their possible role in dental phenotypes. Previous studies have associated genetic polymorphisms in *PTH* with dental dimensional^25^ and genetic polymorphisms in *VDR, CYP24A* and *CYP27B1* with developmental dental anomalies, such as tooth agenesis and microdontia.^23^ Therefore, considering the evidence from previous studies that these genes play an important role in dental development phenotypes, we hypothesized that they could also be involved in dental maturity phenotypes. Thus, the aim of this study was to evaluate the association of dental development (dental maturity) with genetic polymorphisms in *PTH, VDR, CYP24A,* and *CYP27B1*.

## Methods

### Ethical aspects

This study was approved by the Research Ethics Committee of the School of Dentistry of Ribeirão Preto, University of São Paulo (FORP/USP) (CAAE #01451418.3.0000.5419). Informed consent/assent was obtained from all participants and/or from their legal guardians. The Strengthening the Reporting of Genetic Association (STREGA) guidelines were followed.^26^

### Sample characterization

This was a cross-sectional study that used a convenience sample obtained from the orthodontic records of Brazilian children during dental treatment at the Orthodontics Clinic of the of the School of Dentistry of Ribeirão Preto, University of São Paulo, between 2017 and 2018.

### Inclusion criteria

All orthodontic records of male and female children aged 7 to 16 years were included.

### Exclusion criteria

Children with systemic disorders, congenital anomalies, syndromes, and craniofacial deformities were excluded. Children who had undergone previous orthodontic treatment, with a history of dental trauma and bilateral agenesis/missing tooth/teeth, were excluded.^5^

### Phenotype definition - Dental development/dental age analysis

Dental development was evaluated to estimate dental age, and the method proposed by Demirjian, Goldstein, and Tanner was used.^4^ The analysis was carried out using the Dental Age (Crescendo Treinamentos Avançados Ltda, Curitiba, Brazil) mobile application, available for download from various app stores. Dental Age was downloaded on the examiner's cell phone and was used for entering the data according to the method proposed by Demirjian, Goldstein, and Tanner.^4^ This method consists of the panoramic radiographic examination of dental development of all left mandibular teeth, except for the third molars. Scores (A-H) are assigned according to the dental development stage. Therefore, the chronological age of each patient is estimated by matching the sum of the scores with the reference table, according to sex. Dental Age reproduces the method proposed by Demirjian, Goldstein, and Tanner^4^ for the preliminary identification of sex, followed by the identification of scores for each tooth analyzed. At the end of the analysis, dental age is automatically calculated by the application.

After estimating the dental age, a delta [dental age minus chronological age (DA-CA)] was calculated by the examiners. This analysis suggested the patient's tendency towards normal, delayed (negative values), or advanced (positive values) dental maturity.^5,7^ Two observers trained by a senior orthodontist were previously trained and calibrated. The weighted Cohen's kappa test was performed for each evaluated tooth. The intra-observer reliability ranged from 0.82 to 1.00 and the inter-observer reliability ranged from 0.79 to 1.00.

### Genotyping analysis

Genomic DNA for molecular analysis was extracted from cells collected from the saliva.^5,7^ Six genetic polymorphisms with the global minor allele frequency higher than 20% were selected based on previous studies.^19,23^ The selected genetic polymorphisms rs694, rs6256, and rs307247 in PTH and rs7975232, rs464653, and rs927650 in *VDR*, *CYP27B1,* and *CYP27B1*, respectively, were genotyped (Table 1). The examiners were blinded to the patients’ conditions during the laboratory experiment. Genotyping was conducted by real-time polymerase chain reactions (real-time PCR), using the TaqMan assay with the StepOnePlus Real-Time PCR System (Applied Biosystems, Foster City, USA).

**Table 1:** Characteristics of the studied genetic polymorphisms.

Polymorphism	rs694	rs6256	rs307247	rs7975232	rs464653	rs927650
Gene	*PTH*	*PTH*	*PTH*	*VDR*	*CYP27B1*	*CYP27B1*
MAF	0.51	0.12	0.39	0.45	0.24	0.45
Base change	C > T	G > T	G > A	C > A	A > G	C > T
Function	Intron variant	Stop-gained variant	Downstream variant	Intron variant	Intron variant	Intron variant

### Statistical analysis

Maturity (delta DA-CA) was evaluated as a continuous variable. The chi-square test was used to calculate Hardy-Weinberg equilibrium. The Mann-Whitney test was used to compare DA between sexes. The Kruskal-Wallis test was used to compare dental maturity (delta DA-CA) according to genotypes. The statistical analysis was performed using SPSS. The significance level was set at 5% (p < 0.05) for all comparisons.

## Results

Of the 115 initially screened patients, 79 patients were included in this study following the inclusion/exclusion criteria. A total of 35 (44.3%) patients were female and 44 (55.7%) were male. The mean chronological age was 12.57 years (standard deviation = 1.68). The mean dental age was 13.35 years (standard deviation = 1.45). Demirjian, Goldstein, and Tanner's method^4^ overestimated the mean age of patients by 0.75 years (standard deviation = 0.90). In females, the method^4^ overestimated the mean age by 0.77 years (standard deviation = 0.91), while in males, the mean age was overestimated by 0.72 years (standard deviation = 0.90). No statistical difference was observed between delta DA-CA and sex (p = 0.67).

The genetic polymorphisms were in Hardy-Weinberg equilibrium (p > 0.05). Delta DA-CA was compared between the genotypes of each studied genetic polymorphisms (Table 2). None of the genetic polymorphisms were associated with dental maturity (p > 0.05).

**Table 2:** Comparison of Delta DA-CA among genotypes.

Genetic polymorphism	Genotype	n	Mean (SD)	p-value
PTH	T/T	20	0.32 (0.74)	0.125
rs694	C/T	37	0.81 (0.93)	
	C/C	14	0.8 (0.79)	
PTH	G/G	34	0.71 (0.95)	0.640
rs307247	G/A	27	0.64 (0.82)	
	A/A	10	0.61 (0.81)	
PTH	G/G	57	0.61 (0.88)	0.163
rs6256	G/T	12	0.65 (0.60)	
	T/T	1	2.25 (0.00)	
VDR	C/C	32	0.57 (0.96)	0.734
rs7975232	C/A	33	0.89 (0.93)	
	A/A	9	0.82 (0.65)	
CYP27B1	A/A	38	0.81 (0.93)	0.775
rs464653	A/G	30	0.65 (0.98)	
	G/G	7	0.63 (0.52)	
CYP24A1	C/C	24	0.77 (1.04)	0.543
rs927650	C/T	38	0.64 (0.79)	
	T/T	9	0.66 (0.84)	

## Discussion

The comprehensive growth and development of the craniofacial complex is important to establish a balanced relationship between the teeth, the mandible, and other facial structures that participate in occlusion. Thus, studies that investigate dental development are extremely important because of the immense gaps in the existing literature.^1,5,7^ The literature evidences a substantial systemic and genetic contribution to the harmonious development of facial structures.^5,7,27^ *PTH* and vitamin D are widely recognized for their activity in bone metabolism^14-16^ and in dental development.^13,19,22-24,28,29^ The present study aimed to evaluate the association of dental development with genetic polymorphisms in *PTH* and genes involved in vitamin D synthesis (*VDR*, *CYP27B1,* and *CYP27B1*) in a cohort of Brazilian children. Our findings demonstrated no association between the studied variables.

PTH is an 84-amino acid peptide hormone synthesized by parathyroid gland cells. This hormone is an important mediator of bone remodeling, including alveolar bone remodeling,^12^ and plays a crucial role in calcium homeostasis, with several effects on the bone remodeling process, promoting anabolic and catabolic activity.^14,15,29^ Any signs of hypocalcemia lead to a larger release and synthesis of *PTH*, restoring serum calcium to normal levels.^30^ Genetic polymorphisms rs694, rs307247, and rs6256 in the gene encoding *PTH* are functionally involved in the reduction of serum *PTH levels*,^31-33^ which could cause bone dysfunction, altered tooth eruption, and irregular dental root formation.^19,34,35^ In a recent study, genetic polymorphisms rs694 and rs30724 in *PTH* were associated with mandibular retrognathism.^19^ Considering that variations in dental maturity are associated with skeletal malocclusions,^1^ it would be possible to assume that functional genetic polymorphisms rs694, rs307247, and rs6256 in *PTH* could be involved in this process, however, an association was not observed. The findings of the present study may also be related to temporospatial effects on the mediation of distinct fragments of *PTH* and dental development.^36^

Note also that *PTH* is a major stimulator of vitamin D synthesis and activation. Vitamin D represents a group of fat-soluble steroid hormones produced mainly through exposure of the skin to UVB radiation from sunlight. In the skin, 7-dehydrocholesterol is converted into pre-vitamin D_3_ and, in the liver, it is converted into 25-hydroxyvitamin D_3_ (25(OH)D_3_) which, in turn, undergoes hydroxylation and becomes 1,25-dihydroxyvitamin D (1,25(OH)2D_3_), the biologically active form that activates *VDR*. *VDR* is a transcription factor that binds to DNA and regulates hundreds of gene expressions, mediating vitamin D activities and other processes, including cell proliferation, differentiation, angiogenesis, and apoptosis.^37^ Although the biological effects of vitamin D are mediated mainly by its intracellular receptor, other important genes should also be mentioned.^38^ *CYP27B1* catalyzes the hydroxylation of calcifediol to calcitriol (the bioactive form of vitamin D) and *CYP24A1* catalyzes reactions including the 24-hydroxylation of calcitriol. Our findings do not demonstrate an association between dental maturity and polymorphisms in these genes that participate in vitamin D synthesis. Nevertheless, this hypothesis is still highly supported by studies in animal models,^39^ in children with vitamin D-resistant hereditary rickets (a rare genetic disease caused by VDR mutations).^40^

Our findings are restricted to the role of genetic polymorphisms and do not evaluate serum vitamin D and *PTH* levels. Note that, while the studied population lives in the city of Ribeirão Preto, São Paulo, Brazil, where sunlight and UVB incidence are consistent throughout the year, low serum levels of the vitamin D could also be evidenced.^24^ Another limitation of our study is its small sample size. This study represents an initial effort and underscores the need for future research with a larger sample size.

## Conclusion

No association was observed between polymorphisms in *PTH* gene and genes involved in vitamin D synthesis and dental maturity.
